# The role of enteric glia in intestinal immunity

**DOI:** 10.1016/j.coi.2022.102183

**Published:** 2022-08

**Authors:** Fränze Progatzky, Vassilis Pachnis

**Affiliations:** Development and Homeostasis of the Nervous System Laboratory, The Francis Crick Institute, 1 Midland Road, London NW1 1AT, UK

## Abstract

The nervous system and immune system are important interfaces of the gastrointestinal tract that sense, integrate and respond to environmental stimuli and challenges. Enteric glial cells (EGCs), the non-neuronal cells of the enteric nervous system, were long considered mere bystanders only providing support for their workhorse neuronal neighbours. However, work by many groups has demonstrated that EGCs are important nodes in the intestinal tissue circuitry that regulate gastrointestinal barrier function, immunity, host defence and tissue repair. More recent studies have also begun to uncover the cellular interactions and molecular mechanisms that underpin the important functions of EGCs in intestinal physiology and pathophysiology. Here, we review recent literature investigating the roles of EGCs in intestinal immunity and tissue homeostasis.

## Highlights

• Enteric glial cells (EGCs) are required for maintenance of gut tissue homeostasis.
• GFAP+ EGCs regulate the health of the intestinal epithelial barrier.
• EGCs produce immunoregulatory molecules that regulate tissue repair and host defence.

**Current Opinion in Immunology** 2022, **77**:102183

This review comes from a themed issue on **Special Section Neuroimmunology**

Edited by **Roland Liblau** and **Isaac Chiu**

For complete overview of the section, please refer to the article collection, “Special Section Neuroimmunology (June 2022)”

Available online 6th May 2022

https://doi.org/10.1016/j.coi.2022.102183

0952-7915/© 2022 The Authors. Published by Elsevier Ltd. This is an open access article under the CC BY license (http://creativecommons.org/licenses/by/4.0/).

## Introduction

Intestinal immunity is essential for health and survival. Commensurate to its importance, numerous studies have uncovered the multiple contributions of epithelial, immune and stromal cells of the gut in host defence in symbiosis with the microbiota. However, the roles of gut innervation in immunity, and in particular the enteric nervous system (ENS), are less well understood. The ENS, the largest and most complex subdivision of the peripheral nervous system, originates from the neural crest and functions largely independently from the central nervous system (CNS) to control multiple aspects of gastrointestinal function [1]. It is composed of intricate networks of neurons and glial cells which are organised into networks of interconnected ganglia within the gut wall: the myenteric plexus, in the tunica muscularis and the submucosal plexus, beneath the mucosal layer 2, 3. Embedded within the largest immune organ of the body that carries the heaviest load of antigens and microbiota [4], the ENS interacts with the gut immune system, forming neuroimmune cell units that regulate tissue homeostasis and pathogen defence [5]. While functional units between enteric neurons and immune cells are well established 6, 7, 8, 9, the interactions between enteric glial cells (EGCs) and the immune system are only starting to be appreciated.

Ultrastructural and immunohistochemical studies performed four decades ago 10, 11 demonstrated the close association of EGCs to enteric neurons within the ganglia and highlighted their similarities to CNS astrocytes. Since these early reports, a plethora of studies have shown that EGCs have important roles in digestive physiology by supporting myenteric and submucosal neurons and regulating the activity of intestinal neural circuits 12, 13. However, more recent studies, partly motivated by the realisation that EGCs are not restricted to the confines of the ganglia but are distributed widely throughout the intestinal wall, have shown that beyond their ‘canonical’ neuroprotective and regulatory functions, enteric glia make critical contributions to the broader tissue physiology in the gut. Here, we highlight recent studies that uncover how the crosstalk between EGCs and other cell types within the intestine, such as intestinal epithelial cells and cells of the immune system, regulates homeostasis, host defence and tissue repair.

## Enteric glial cells regulate the health of barrier tissues in the intestine

The notion that EGCs directly promote the fitness of the intestinal epithelial barrier dates back to studies performed more than 20 years ago, when three groups reported independently that ablation of EGCs in mice leads to severe intestinal inflammation 14, 15, 16. Using different ablation approaches, such as administration of a gliotoxin [14], injection of ganciclovir to transgenic mice expressing herpes simplex virus thymidine kinase under the control of the Glial Fibrillary Acidic Protein (*GFAP)* promoter [15] and autoimmune targeting of EGCs [16], these groups demonstrated that loss of enteric glia resulted in vascular lesions and disrupted epithelial integrity leading to severe intestinal inflammation. Subsequent reports confirmed and extended these findings by demonstrating that targeting GFAP+ EGCs led to increased intestinal epithelial barrier permeability, impaired epithelial cell proliferation and recruitment of immune cells into the ganglia 17, 18, 19. However, a more recent report showed that depletion of EGCs by diphtheria toxin expressed under the control of the proteolipid protein 1 (PLP1) promoter did not affect epithelial cell renewal or permeability and did not alter the susceptibility of mice to dextran sodium sulphate (DSS)-induced colitis [20]. Although one cannot exclude the possibility that some of these differences could be related to how faithfully transgenic tools reproduce the patterns of expression of the corresponding endogenous gene, recent work has provided evidence that the population of EGCs encompasses functionally heterogeneous groups of cells distinguished by the expression of commonly used molecular markers [21]. Specifically, these authors demonstrated that while diphtheria toxin-induced ablation of PLP1+ EGCs was remarkably innocuous, targeting of the GFAP+ subset of EGCs impaired the function and regenerative response of epithelial stem cells [21]. Interestingly, simultaneous ablation of both populations of EGCs resulted in a severe collapse of intestinal tissue architecture and lethality after only a few days [21]. At first sight, the critical role of the GFAP+ EGCs in the maintenance and dynamics of the intestinal epithelium contrasts with the observation that only a relatively small percentage of enteric glia expresses high levels of GFAP at steady state 22, 23. However, the rapid upregulation of GFAP expression observed in response to inflammation 24, 25, 26, 27••, together with the ablation studies, argue for a dynamic relationship between EGC subpopulations and suggest that the PLP1+ subset replenishes the GFAP+ pool following its ablation. In support of the plasticity of enteric glia and the interrelationship of its subpopulations, EGCs in the intestinal mucosa of mice are continuously replenished by their myenteric counterparts in response to the microbiota [28]. Which specific signals mediate the shift from PLP1+ to GFAP+ EGC-states and whether microbial factors are involved remains to be determined.

Enteric glia have emerged as a signalling hub capable of responding to environmental and tissue changes by producing diffusible factors that modulate the behaviour of neighbouring cell types 29, 30. Among such glia-derived signalling cues, recent evidence has suggested that Wingless and Int-1 (WNT) ligands produced by GFAP+ EGCs regulate cellular turnover in the intestinal epithelium [21]. This idea is supported by both gene expression analysis and genetic experiments in which inhibition of WNT ligand secretion by GFAP+ EGCs resulted in a failure to support intestinal stem cells (ISCs) [21]. Interestingly, other cell types in the mucosa, including stromal cells, are also rich sources of WNT ligands [31], suggesting that GFAP+ enteric glia establish a ‘special relationship’ with epithelial cells that cannot be reproduced by other non-glial cell types. In support of this, several groups have shown that EGCs form basket-like structures surrounding the base of intestinal crypts (where ISCs are located) 28, 32, 33, 34, establish direct contacts with enteroendocrine cells [35] and form intimate associations with colorectal cancer cells [36]. It appears therefore that this privileged position of EGCs relative to the intestinal epithelial layer allows signals secreted by EGCs to serve as effective regulators of epithelial cell dynamics and barrier fitness in the intestine.

The epithelium of the colon is one of the most frequent sites of tumour initiation while hyperactivation of the WNT signalling pathway has been implicated in the pathogenesis of colorectal cancer [37]. Given the emerging effects of glia-derived WNT ligands on the intestinal epithelium, it is interesting that depletion of GFAP+ EGCs led to a striking reduction of tumour burden in a model of azoxymethane/DSS-induced colon cancer that was much less obvious following depletion of PLP1+ EGCs [38]. Although it is unclear whether glia-derived WNTs are implicated in tumour formation in this model, together, these studies support the central role of the GFAP+ (but not the PLP1+) subpopulation of EGCs in intestinal epithelial health 20, 21••, 38•. In further support of a regulatory circuit involving enteric glia and the intestinal epithelium, a functional unit has been previously described in which GFAP+ EGCs produce glia-derived neurotrophic factor (GDNF), in a myeloid differentiation primary response 88 (MYD88)-dependent manner, that acts on rearranged during transfection (RET)-expressing type 3 innate lymphoid cells to secrete the tissue-protective cytokine IL-22 [39]. GDNF-induced IL-22 regulates intestinal epithelial barrier protection and defence during DSS-induced colitis and *Citrobacter rodentium* infection via production of antimicrobial peptides and mucins [39]. Irrespective of whether the effects of EGCs on the maintenance and repair of the intestinal epithelium are direct or indirect (via other cell types), these recent studies strengthen the view that EGCs have crucial roles to the pathogenesis of diseases associated with intestinal epithelia barrier dysregulation, such as inflammatory bowel disease (IBD) or cellular transformation, such as colon cancer [60]. These findings provide renewed urgency to the possibility of harnessing EGCs and their effectors as therapeutic targets for these common conditions. Therefore, understanding the cellular and molecular foundations for the ‘barrier-protective function’ of GFAP+ EGCs is a fascinating area for future investigation, both in terms of fundamental biology and translational science.

The intestinal epithelium is not the only barrier tissue enteric glia are associated with. Similar to the glia limitans in the brain that maintain the blood brain barrier [40], EGCs are closely associated with intestinal blood vessels, suggesting a critical role of these cells in the maintenance of the gut-vascular barrier 28, 33, 41. A similar anatomical and functional relationship can be hypothesised also for EGCs and the closely associated lymphatic endothelial cells [42]. Also, similar to their role in supporting the epithelium on the luminal side of the intestine, EGCs are likely to be crucial components of the niche that regulates the homeostasis and activation of the mesothelium, a single cell layer that lines the surface of the intestine and is fundamental to the maintenance of serosal integrity [43]. Using a conditional knock-out mouse model in which EGCs were unable to respond to the inflammatory cytokine interferon gamma (IFNγ), we demonstrated recently that mesothelial cells exhibit activated morphology and overexpress inflammatory markers at both steady state and in response to pathogens [27]. Whether glia-derived WNT ligands are also responsible for the changes in mesothelial cells observed in this mouse model or whether the mesothelium responds to more global changes in their cellular environment requires further investigation. Irrespective of the mechanisms, the emerging roles of enteric glia in the maintenance of the intestinal epithelium, the serosal mesothelium and potentially the vasculature of the gut wall, argue that EGCs have adopted central roles in safeguarding tissue integrity along the entire luminal-serosal axis of the gut wall. Whether and how glial cells in other visceral organs have barrier protective roles similar to those of EGCs is an exciting question for future studies.

## Immunomodulatory roles of enteric glial cells

EGCs have long been thought to have immunoregulatory effects in intestinal inflammatory conditions due to their similarities with immune cells, both in terms of molecular make up and observed responses. Thus, EGCs are able to sense microorganisms (and other damage-associated molecular patterns) and immune-cell derived signals through the expression of functional pattern recognition receptors (e.g. toll-like receptors — TLRs), cytokine receptors (e.g. interferon-gamma receptor — IFNγR) and the associated machinery to respond to inflammatory stimuli (e.g. Nuclear factor kappa B — NF-κB, MYD88, signal transducer and activator of transcription — STAT1, STAT3, etc.). Furthermore, upon stimulation, EGCs secrete cytokines and chemokines implicated in pro- or anti-inflammatory activation 12, 30, 44, 45, 46. While the diverse responses of EGCs to inflammatory stimuli have been characterised mostly *in vitro* 30, 44, it is becoming increasingly clear that EGCs can respond to tissue challenges *in vivo* and adopt distinct molecular states characterised by cell cycle entry and GFAP upregulation 12, 24, 25, 26, 27••, which are reminiscent of astrogliosis [47] and mediate local immunomodulatory effects on neighbouring cells 27••, 39, 48••, 49•. Reactive EGCs have been detected in patients with IBD 16, 50, although it is still unclear whether this is the cause or a consequence of inflammatory disease progression. In that respect, it is interesting that EGCs isolated from patients with ulcerative colitis display an IFNγ gene signature that is also detected in the activated cell state of mouse EGCs after parasite infection [27]. Disruption of the IFNγ–EGC signalling axis during helminth infection in mice enhances the inflammatory response and impairs tissue healing [27]. These findings, together with the recent demonstration that EGCs are involved in a mouse model of necrotising enterocolitis [51], suggest that mammalian enteric glia are implicated in the pathogenesis of common conditions associated with dysregulated immunity, such as IBD. A better understanding of the molecular mechanisms underpinning the crosstalk between EGCs and immune cells will be critical for elucidating the role of enteric glia in maintaining intestinal health.

One example of such interaction is the EGC-muscularis macrophage axis. Both at steady state and in response to parasite (helminth) invasion of the small intestine, EGCs control the level of activation of muscularis macrophages (MMs) via IFNγR-dependent mechanisms [27]. Also, in response to muscularis damage, EGCs stimulate monocyte recruitment via chemokine (C-C motif) ligand 2 and modulate their differentiation towards pro-resolving macrophages via macrophage colony-stimulating factor (M-CSF) [52]. In contrast, in a mouse model of colitis, EGCs drive pro-inflammatory MM activation via M-CSF production contributing to long-lasting visceral hypersensitivity [48]. Of note, EGCs have been shown to be the predominant source of M-CSF production in the muscularis [48] and it will be important to determine whether this cytokine is also responsible for the anti-inflammatory effects of EGCs during intestinal immune homeostasis [27]. The mechanisms underpinning the inflammatory context-dependent functions of EGC-derived M-CSF on MMs and recruited monocytes in vivo and roles of particular EGC subpopulations in regulating MMs within subdomains of the complex tissue landscape of the tunica muscularis, remain unknown.

In addition to their role in innate immunity, there is compelling evidence that EGCs also regulate the recruitment and activation of adaptive immune cells. For example, in co-culture experiments, EGCs are capable of suppressing T-lymphocyte proliferation [53]. Further, a recent report has provided evidence that EGCs function as antigen-presenting cells that modulate T-lymphocyte and B-lymphocyte activation [49] through the upregulation of class II major histocompatibility complex molecules (MHCII) 54, 55, 56. During helminth infection and in response to increased levels of IFNγ, EGCs upregulate C-X-C motif chemokine ligand 10 (CXCL10) to recruit IFNγ^+^CD8^+^ T cells to the tunica muscularis in order to amplify the IFNγ tissue response and propagate immune cell activation [27]. Of note, CXCL10 is already expressed at very low levels in EGCs at steady state and is rapidly induced upon infection or in response to IFNγ [27]. This observation echoes the rapid induction (within hours) of CCL2 and M-CSF expression following injury of the tunica muscularis [52]. Finally, MHCII expression in EGCs is induced in response to low levels of IFNγ in the absence of inflammation or tissue damage [49], and MHCII is detected on EGCs from macroscopically normal areas of intestinal biopsies of Crohn’s disease patients and occasionally in healthy individuals 54, 56. Together, these observations indicate that EGCs are equipped with the molecular machinery to monitor the inflammatory state of the intestine and are among the first cells to be mobilised in a cellular cascade that leads to the recruitment and activation of effector immune cells capable of clearing an infection and repairing tissue damage.

## Concluding remarks

**Figure 1:**
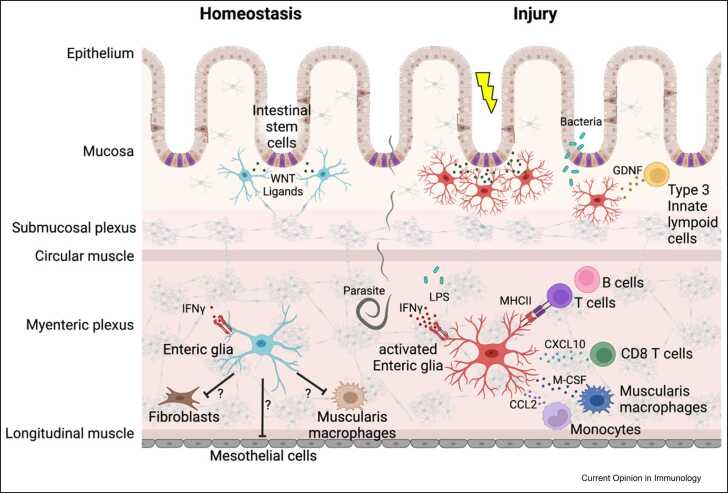
Functional units of enteric glia and other cell types in the gut control immune homeostasis and the response to infectious and inflammatory challenge. Depicted on the left are the homeostatic roles of enteric glial cells (light blue) in maintaining the intestinal epithelium and the inflammatory tone of the tunica muscularis. In response to pathogen invasion (e.g. helminth parasites), inflammation or tissue injury (e.g. irradiation), enteric glial cells are activated (red) and produce a range of diffusible factors or express cell-surface molecules to control immune responses and tissue repair. CCL2: chemokine (C-C motif) ligand 2, LPS: Lipopolysaccharide, Cxcl10: C-X-C motif chemokine ligand 10, M-CSF: macrophage colony-stimulating factor, MHCII: major histocompatibility complex class II, IFNγ: interferon gamma, GDNF: glia-derived neurotrophic factor. Figure created with BioRender.com.

EGCs have emerged as a cell type that is fully integrated into the tissue environment of the gut and continues to surprise us with new discoveries revealing the different ways in which these cells contribute to the regulation of digestive physiology and host defence. The studies highlighted here (Figure 1) add to the growing evidence of morphological 23, 57 and molecular [27] 58••, 59 heterogeneity of EGCs, and provide further support to the view that this cell population is functionally diverse. Understanding how enteric glia are recruited into diverse physiological roles and how such specialised activities may be conscripted during pathology is an exciting area for future research. In particular, it will be interesting to determine whether the immune function of EGCs is controlled by lineage-specific gene expression programmes or signals from the tissue or luminal (diet or microbiota) environment of the gut. Understanding the cell-intrinsic mechanisms and environmental cues that empower EGCs with immunoregulatory functions will help us harness these cells for the treatment of conditions associated with immune dysregulation of the gut. Given the profound effects of glial cells on neuronal activity [12], it will also be important to explore further the regulatory roles of EGCs on the synthesis and secretion of diverse immunomodulators by enteric neurons. We anticipate that over the forthcoming years enteric glia can serve as an excellent model for understanding the roles of other populations of peripheral or CNS glia in tissue homeostasis and immune responses. Current advances in molecular profiling of EGCs in the context of the diverse tissue milieu of the intestine promise to deliver much-needed tools to the research community for the study of these cells in health and disease.

## Conflict of interest statement

Nothing declared.
